# Successful treatment of renal cell carcinoma with paraneoplastic nephrotic syndrome: a case report

**DOI:** 10.3389/fmed.2025.1506592

**Published:** 2025-03-27

**Authors:** Arshdeep Singh Singh, Javier Naranjo, Marta Alonso, Florentino Villanego, Jose Manuel Amaro, Daniel Carrasco, Cristina Salvatierra, Auxiliadora Mazuecos

**Affiliations:** ^1^Department of Nephrology, Puerta del Mar University Hospital, Cadiz, Spain; ^2^Department of Pathological Anatomy, Puerta del Mar University Hospital, Cadiz, Spain; ^3^Department of Urology, Puerta del Mar University Hospital, Cadiz, Spain

**Keywords:** membranous nephropathy, nephrotic syndrome, papillary renal cell carcinoma, nephrectomy, paraneoplastic nephropathy, case report

## Abstract

The association of renal cell carcinoma (RCC) with paraneoplastic membranous nephropathy (MN) is rare. Accurate identification and treatment of the primary tumor can lead to resolution of nephrotic syndrome. We report the case of a 72-year-old male was referred to Nephrology due to significant proteinuria (Urine albumin-creatinine ratio 14,000 mg/g) without clinical nephrotic syndrome. Imaging revealed a nodular lesion in the left kidney, compatible with the diagnosis of papillary renal neoplasia. A total left nephrectomy confirmed papillary RCC and MN. Post-surgery, renal function initially declined but stabilized, with proteinuria significantly reduced by the latest follow-up. Review of 20 cases of nephrotic syndrome associated with RCC revealed that MN was the most frequent underlying nephrosis. MN is frequently associated with solid tumors, but its link with RCC is uncommon. In our review, clear cell carcinoma (CCC) was the most prevalent RCC subtype linked with MN. Early cancer screening in MN patients can uncover occult malignancies, facilitating timely treatment. RCC-associated MN is rare but should be considered in patients presenting with nephrotic syndrome. Surgical resection of the RCC can lead to resolution of the associated nephropathy. This case underscores the importance of thorough cancer screening in patients with unexplained nephrotic syndrome.

## Introduction

The concept of paraneoplastic glomerulopathy was introduced in 1922 by Galloway (1) and refers to clinical manifestation of a renal disease that appears to be secondary to malignant tumors and may be caused by secretion of cancer-cell products. An association between MN and cancer was described decades ago by Lee et al. (2) and later was reported frequently.

Membranous nephropathy (MN) is one of the most frequent causes of nephrotic syndrome in adults and the elderly, while it is rare in children and adolescents. It is a disease caused by antibodies directed against various podocyte antigens causing podocyte damage and the deposition of immune complexes on the outer surface of the glomerular basement membrane (GBM). In secondary MN the pathogenic mechanism is also caused by the deposition of immune complexes at the same site, but in this case the antigens are associated with systemic diseases, tumors, infections or various drugs (3). The frequency of cancer in the MN population has been variously estimated at between 5 and 22%, lung carcinomas being the tumors most frequently associated with MN, followed by prostate carcinomas. The association with renal cell carcinomas (RCC) is rare (3).

Accurate identification of the cause is essential, as specific treatment of the cause (e.g., resection of the tumor) leads in many cases to resolution of the nephrotic syndrome.

## Case report

We report the case of a 72 years old Caucasian male with previous history of benign prostatic hyperplasia, Menière’s syndrome, hypertension under treatment with enalapril, dyslipidemia, obesity and ex-smoker who was referred to Nephrology in June 2021 due to 14,000 mg/g of proteinuria measured by urine albumin/creatinine ratio (UACR), with overall good condition and no other complaints or symptoms. He had no known allergies, no prior surgical history, and no significant family history of medical relevance. Serum creatinine (SCr) was 0.8–1.1 mg/dL. Glomerular filtration rate was 68 mL/min. Physical examination revealed a blood pressure of 115/70, with no edema in both lower extremities. Laboratory examination findings were as follows: albumin 3 g/dL, total cholesterol 267 mg/dL, triglycerides 238 mg/dL. Antinuclear antibody, anticytoplasmic antibody and anti-PLA2r were negative; immunoglobulin, complement 3 and complement 4 were normal. NELL1 and THSD7A assays are not available in our center.

**Figure 1:**
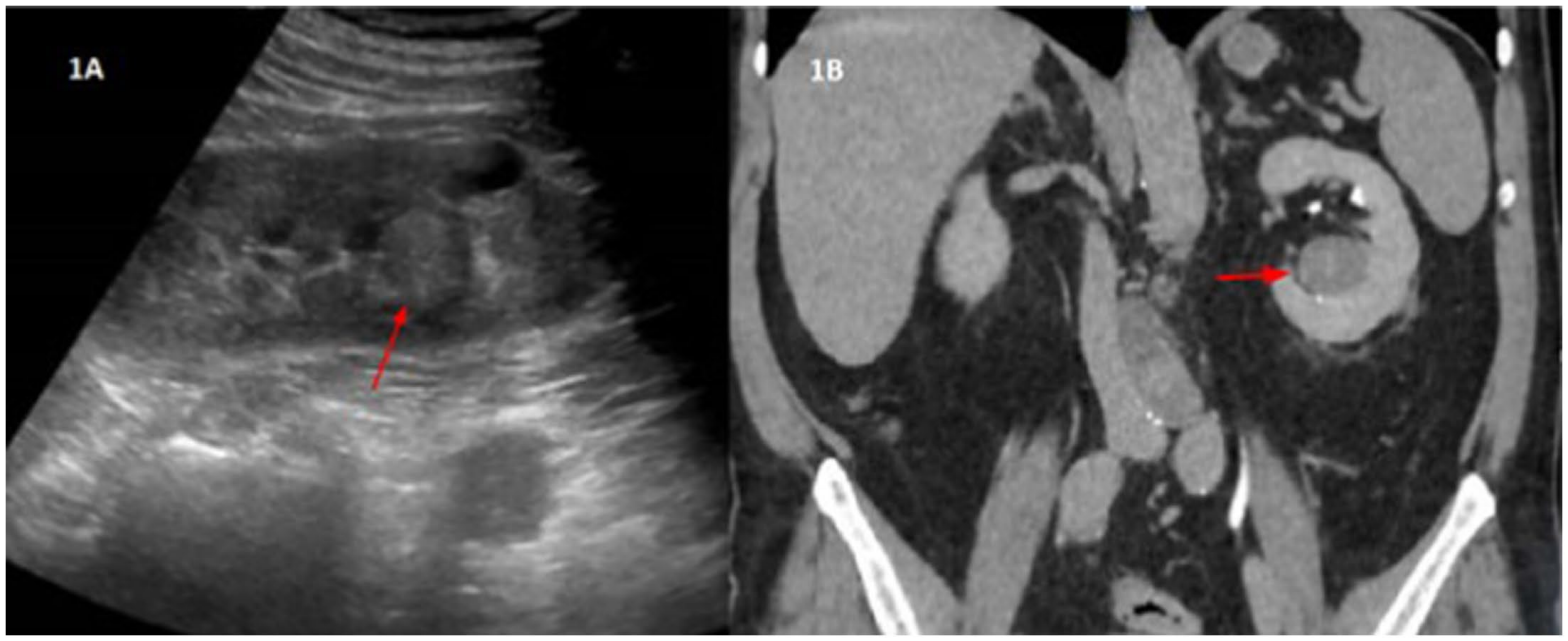
**(A)** Ultrasound imaging. Nodular image in the left renal sinus, of approximately 2 cm, isoechogenic with the cortex. **(B)** Uro-CT-Scan. In the lower portion of the left renal sinus, a nodular lesion, isodense with the renal cortex, is seen. It has homogeneous density, with rounded morphology and well-defined edges. Approximate measurements by CT are of 3.3 × 2.9 × 2.7 cm in the transverse, anteroposterior and craniocaudal axes, respectively. This lesion shows a density of 46 HU, therefore it is a solid lesion. In the portal phase it presents 56 HU, that is, it shows very slight uptake. In the excretory phase, the lesion has a density of 67 HU, which confirms a slowly progressive uptake. These findings are suggestive of papillary renal neoplasia as the first diagnostic option.

An ultrasound exam showed a nodular image of approximately 2 cm in the left renal sinus described as isoechogenic with the cortex. We completed the study with a computed tomography urography, which suggested probable papillary renal neoplasia (Figure 1) without other relevant findings in the study. The case was discussed with urology and a partial nephrectomy was ruled out due to the location of the tumor. A total left nephrectomy was performed in June 2022. The patient was positioned in a right lateral decubitus with forced flank elevation, and access was achieved via a left paraumbilical optical trocar placed slightly superior to standard positioning. Additional working trocars were inserted along the anterior axillary line: a 5 mm subcostal and an 11 mm above the right iliac crest, with an infraumbilical auxiliary trocar. No adhesions were present. The left colon was mobilized to expose the ureter at its crossing with the iliac vessels, followed by isolation of the renal artery and vein, which were individually clamped and secured using *Hem-o-lok Polymer Ligation System* Clips (3 proximally, 1 distally). The upper pole of the kidney was freed while preserving the adrenal gland. The ureter was dissected, clamped with *Hem-o-lok Polymer Ligation System* Clips, and divided with a *LigaSure* vessel sealing device. A 10 cm extension of the auxiliary trocar incision was made for specimen extraction. Hemostasis was confirmed, a 19 ch Blake Style Surgical Silicone Drain was placed, and the wound was closed in a single layer using continuous No. 1 *MonoPlus* long term loop synthetic absorbable monofilament suture, with skin closure achieved via staples. Total operative time was 130 min, with an estimated blood loss of less than 100 cc. The surgical procedure was completed without any complications.

**Figure 2:**
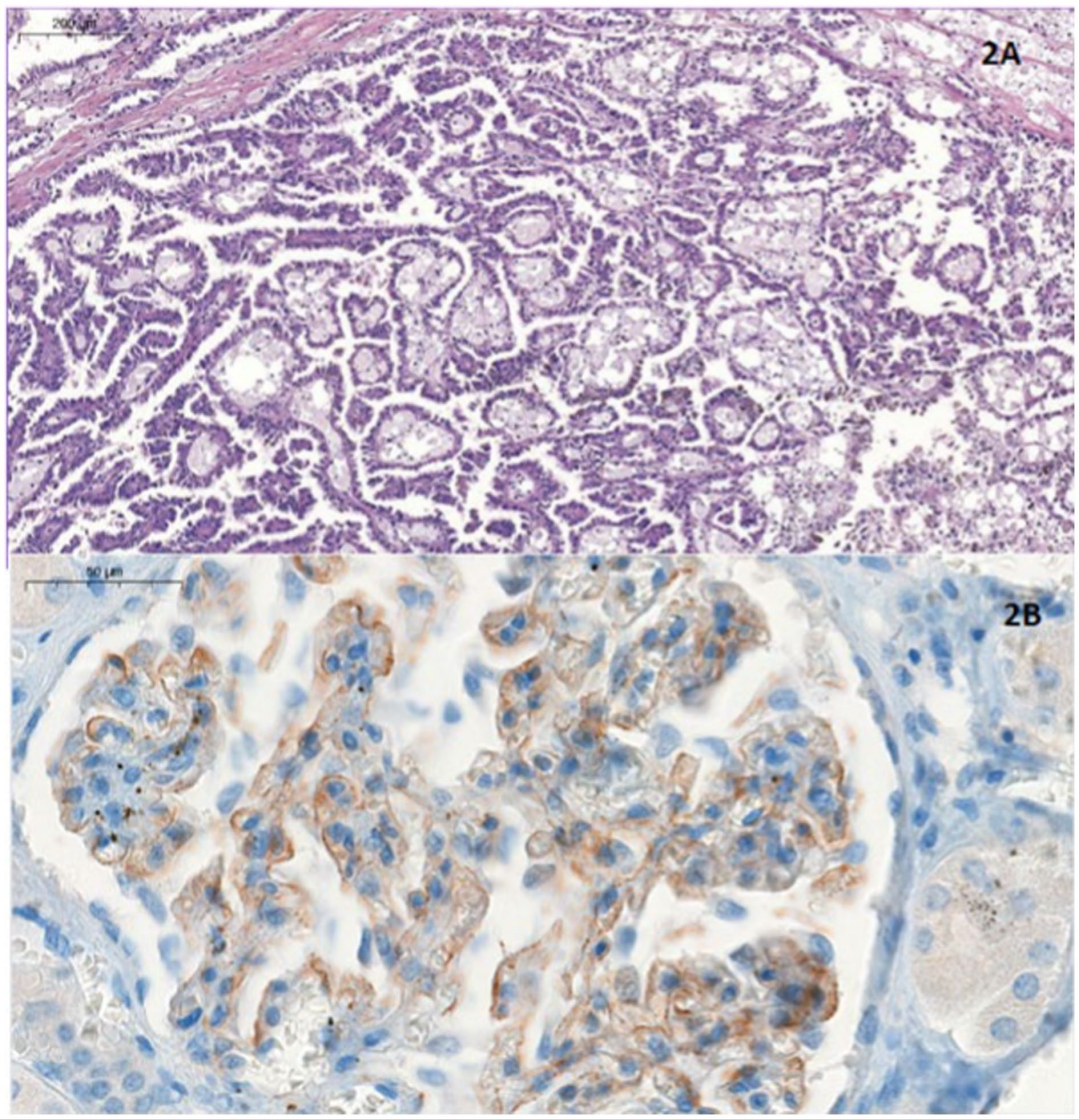
**(A)** Hematoxylineosin stain. Papillary structures, lined with eosinophilic cuboidal cells, some with vacuoles. Foamy macrophages and haemosiderin deposits are present. All these findings are compatible with papillary carcinoma. **(B)** Immunohistochemical analysis for C4d. At higher magnification we see granular deposits which are C4d deposits. This is consistent with the diagnosis of membranous GMN type 1.

In the nephrectomy specimen, a unifocal renal papillary carcinoma of 2.5 cm in lower pole (pT1aNx) was diagnosed. In addition, renal techniques hematoxylin-eosin, Schiff’s Periodic Acid, Silver-methenamine, Masson’s trichrome and Congo Red and immunohistochemistry (IH) were performed on the rest of the non-tumor renal parenchyma, showing global sclerosis of 10% of the glomeruli and membranous granular deposits of C4d in the IH study, with negative results for IgG, IgG4, kappa and lambda. It was not possible to perform immunofluorescence or examination with an electron microscope. Given these data, a diagnosis of stage I membranous glomerulonephritis was made (Figure 2).

After surgery, blood tests showed impaired renal function (SCr 1.7–2 mg/dL). An ultrasound and scintigraphic study were performed, with no relevant alterations. In the latest check-ups, renal function has remained stable at SCr 2 mg/dL and proteinuria has progressively decreased, with the last determination at UACR 31.5 mg/g in February 2024. The patient is currently asymptomatic, maintains an active lifestyle, and has no history of additional hospitalizations.

## Discussion

The association of nephrotic syndrome and RCC is rare (4). We review 20 cases of nephrotic syndrome associated with RCC (Table 1) (4–16). The association of nephrotic syndrome with RCC and nephrosis secondary to systemic diseases such as vasculitis or metastatic disease is not studied in this review. MN is one of the most frequent causes of nephrotic syndrome. In half the patients (50%) of our review the underlying nephrosis was MN, followed by minimal change disease in 25% of the patients, membranoproliferative glomerulonephritis (10%), mesangiocapillary glomerulonephritis (5%) and crescentic glomerulonephritis in one patient (5%). In one case (5%), the underlying histopathological data of the nephrosis was not reported.

**Table 1:** Literature review of the underlining diagnosed nephrosis in 20 renal cell carcinoma cases with predominantly nephrotic syndrome and no systemic disease related nephrosis to date.

References	Sex	Age	Renal tumor type	Tumor size (cm)	Surgery	Nephrosis	Serum albumin (g/dL)	Proteinuria (g/24 h)	GFR (mL/min/1.73 m^2^) at diagnosis	Outcome
(4)	M	63	Papillary	N/A	N/A	MN	1.8	24	43	N/A
(4)	M	62	N/A	7 × 6.5	Radical	MN	N/A	N/A	N/A	Nephrosis recovery
(4)	F	77	CCC	12	Radical	MN	1.7	14.9	61	Nephrosis recovery
(4)	M	58	CCC	5	Radical	MN	N/A	N/A	N/A	N/A
(4)	F	76	N/A	4	Radical	MN	1.8	4.4	62	Death due to heart disease
(4)	M	62	N/A	1.6	Partial	MN	2.6	5.3	40	Nephrosis recovery
(4)	M	55	N/A	N/A	Radical	MN	N/A	N/A	N/A	Nephrosis recovery
(4)	F	69	CCC	2 × 2	Partial	MCD	2.8	5.2	100	Nephrosis recovery
(5)	F	64	Papillary	7 × 7	Radical	MCD	2.4	20.7	23	N/A
(6)	M	55	Adenocarcinoma	N/A	None	MG	2.5	++++ (qualitative)	N/A	Death (1)
(7)	M	49	Oncocytoma	7	Radical	MCD	3.09	++++ (qualitative)	86	Nephrosis recovery
(8)	M	70	CCC	2.5	Radical	MCD	N/A	18.8	N/A	Nephrosis recovery (2)
(9)	M	78	Adenocarcinoma	3.5 x 3 x 2.5	Nephrectomy	MCD	3	4.8	22	Nephrosis recovery
(10)	M	55	Papillary	12 x 9 x 6	Radical	Unknown	N/A	3.8	N/A	N/A
(11)	M	65	CCC	6 x 6 x 2	Radical	MPGN	3.3	9.4	19	Nephrosis recovery
(12)	M	42	CCC	3.5 × 3.5 × 5.5	Partial	Crescentic	3.7	4	30	HD
(13)	F	72	CCC	3 × 2.9 × 2.9	Partial	MN	3.8	5.3 (ACR)	28	Nephrosis recovery
(14)	M	57	CCC	6	Radical	MN	2.3	5	60	Nephrosis recovery (3)
(15)	M	65	N/A	4.7 × 7.3	Radical	MPGN	3.2	2.4	37	Nephrosis recovery
(16)	M	68	CCC	5	Embolization	MN	1.1	8.3	40	Death (4)

ACR, albumin/creatinine ratio; CCC, Renal clear cell carcinoma; GFR, glomerular filtration rate; HTA, hypertension; MN, Membranous nephropathy; MCD, Minimal change disease; MG, mesangiocapillary glomerulonephritis; MPGN, membranoproliferative glomerulonephritis; N/A, not available; RCC, renal cell carcinoma. (1) Patient also had leukemia. (2) After the biopsy the patient’s urinary protein excretion declined rapidly and was undetectable 8 days later, no treatment having been given. The left kidney was removed 12 days after the biopsy. (3) With posterior relapse and metastatic progression. (4) Patient also had esophageal adenocarcinoma. Urology felt he was a suboptimal candidate for nephrectomy. The patient ultimately decided to forgo any additional surgery and was discharged to hospice.

It has been well documented in the literature that MN is closely associated with solid tumors. Lung cancer has been the most commonly associated solid tumor, accounting for almost a quarter of all cancer patients, followed by prostate cancer, colorectal cancer, breast cancer and stomach and esophageal cancer, respectively. However, the association with RCC is rare (3). The type of RCC most linked to nephrotic syndrome, as well as the sequence of their association, remains unclear. According to our findings, the most prevalent association of nephrosis is with clear cell carcinoma (CCC) (45%), followed by papillary tumor (15%), adenocarcinoma (10%) and one patient had oncocytoma (5%). Histopathological data was not reported in five studies which could be a limiting factor. Further analysis, as more cases are reported, should be conducted to better understand the prevalence. In the case of MN, 5 of the total of 10 reported cases of MN were CCC (50% of MN), followed by 4 cases where no histopathological data was available and 1 papillary tumor (10% of MN).

Nephrotic syndrome is occasionally a marker of occult solid tumors and hematologic malignancies, with a more than 4% risk in the first year after nephrosis diagnosis. For patients with MN, as reported by Leeaphorn et al. (3), approximately 10% of MN patients have the diagnosis of cancer before the diagnosis of MN. In the case of RCC, no patients in our review had the diagnosis of RCC before the diagnosis of MN or any other nephrosis. Cancer was discovered at the time of or following the diagnosis of MN in the remainder in all cases. This finding is important because it emphasizes the existence of a window after the diagnosis of nephrosis when the patient can benefit from cancer screening in comparison to non-kidney cancers where the diagnosis of cancer could precede that of the MN.

The precise pathogenic mechanism whereby cancer might be associated with MN has yet to be elucidated. The most accepted mechanism is that the antigens involved (from infectious, pharmacological, tumor or other sources) are first deposited between the GBM and podocytes; specific antibodies generated against these antigens cross the GBM to couple with them, leading to the *in situ* formation of immune complexes. A distinction between idiopathic and secondary MN can be made more precisely by the detection of circulating antibodies against PLA2R (17). In general, it is accepted that anti-PLA2R positivity is always diagnostic of primary NM, even if the patient has other conditions (e.g., tumors or infectious diseases) that could theoretically be responsible for the process. Cases of primary MN associated with positive anti-PLA2R have been described in patients with various tumors in which proteinuria does not change after removal of the tumor, reflecting that the tumor was not the cause of the renal disease (17).

In addition to PLA2R, other autoantigens have been identified in primary MN, including thrombospondin type 1 domain-containing 7A (THSD7A) and neural epidermal growth factor-like 1 (NELL1). NELL1-associated MN is a distinct kidney disease characterized by the overexpression of NELL1 in podocytes. It is the second most common target antigen after PLA2R, accounting for approximately 5–10% of all MN cases after excluding autoimmune conditions like lupus. THSD7A, an endogenous antigen expressed on podocytes, accounts for 1–3% of all MN cases. Circulating anti-THSD7A antibodies have been detected in some cases as a result of THSD7A expression by malignant tumors. Both NELL1 and THSD7A have been associated with MN secondary to malignancy. Studies suggest that 10–33% of patients with NELL1-associated MN have an underlying malignancy, compared to 16% of patients with THSD7A-associated MN, highlighting the importance of thoroughly evaluating potential malignancies in these cases (18, 19). Unfortunately, our center does not have access to testing for NELL1 and THSD7A.

It has now become standard practice to search for malignancy in older patients with newly diagnosed MN once other secondary causes have been excluded. However, there is no consensus on how aggressive clinicians should be in search of occult malignancy. In the absence of internationally recognized guidelines and evidence-based indications, expert opinion (20) recommends vigorous pursuit for cancer in a patient who is diagnosed with MN but has no telling serologic features (anti-PLA2R, ANA, hepatitis B, and so forth). Our findings also associate RCC with other nephrosis, therefore cancer screening in all patients with nephrosis, including MN, should be pursued.

As per the treatment options, the 2022 European Guidelines on RCC recommend surgery in localized RCC, with partial nephrectomy (PN) preferred for localized T1 RCCs (less than 7 cm) over radical nephrectomy (RN), although the localization is also considered. Embolization is suggested as a beneficial palliative intervention. Treatment decisions for frail patients should be individualized, weighing the risks and benefits of PN versus RN, the higher risk of perioperative complications, and the risk of developing or worsening chronic kidney disease postoperatively (21). After a nephrectomy, kidney function can deteriorate due to the loss of renal mass, as seen in our patient. Studies have shown that this decline is approximately 6.5 times greater following a RN compared to a PN over a two-year follow-up period (22). Follow-up should include renal function monitoring and also recurrence surveillance (21).

According to the 2022 European Guidelines on RCC, no consensus exists on surveillance schedules after treatment, though expert opinions suggest tailoring follow-up based on proposed risk profiles. Evidence does not show that early diagnosis of recurrences improves survival. Outcomes for T1a low-grade tumors are generally excellent. Patients with a positive margin after partial nephrectomy require closer monitoring due to higher recurrence risk, with type of surgery performed and histological RCC subtype also influencing recurrence risk (21). In our case, our patient had low-risk of recurrence and he underwent monthly follow-ups with quarterly CT scans for 6 months, transitioning to biannual visits and annual CT scans at present with no evidence of recurrence.

In our findings, although only six RCC cases were larger than 7 cm and classified as T1, radical nephrectomy was performed in 12 cases, potentially influenced by individual characteristics. Surgery was not performed in two patients: one declined the procedure, and the other had comorbidities, leading to a preference for embolization. There is no data available on the management of one case. In our case, the surgical team preferred radical nephrectomy due to the tumor’s location in the renal sinus.

In our review, more than half of the patients (60%) showed nephrosis recovery during follow-up after tumor removal. Three patients died (15%), one of them due to a heart disease, one due to a concomitant esophageal tumor and the third patient also had leukemia. One patient (5%) entered hemodialysis. There is no data on the follow-up evolution of the remaining four patients.

In cases of nephrotic syndrome with no identifiable cause, particularly in instances of MN known to be associated with solid tumors, it is imperative to investigate for occult malignancies, although the occurrence of renal cancer remains a rare possibility.

## Conclusion

There is a rare association between RCC and MN as a paraneoplastic syndrome. Our study underlines the importance of ruling out the presence of cancer in patients with nephrotic syndrome. Surgical excision of the RCC may resolve the associated paraneoplastic nephropathy, underscoring the importance of tumor removal in these patients.

## Data Availability

The raw data supporting the conclusions of this article will be made available by the authors, without undue reservation.
